# Clinical Importance of Cannabinoid Type 1 Receptor (CB1R) and Cannabinoid Type 2 Receptor (CB2R) Expression in Renal Cell Carcinoma

**DOI:** 10.7759/cureus.55121

**Published:** 2024-02-28

**Authors:** Dimitrios Deligiannis, Ioannis Anastasiou, Dionysios Mitropoulos, Panagiotis Mitsos, Stamatios Theocharis

**Affiliations:** 1 Department of Urology, Naval Hospital of Athens, Athens, GRC; 2 First Department of Urology, Medical School, Laiko Hospital, National and Kapodistrian University of Athens, Athens, GRC; 3 Department of Urology, Agia Sofia Children's Hospital, Athens, GRC; 4 First Department of Pathology, Medical School, National and Kapodistrian University of Athens, Athens, GRC

**Keywords:** renal cancer prognosis, cannabinoid receptors, carcinomas renal cell, cb2 receptor, cb1 receptor

## Abstract

Background and objective

The purpose of our study was to assess the expression of cannabinoid type 1 receptor (CB1R) and cannabinoid type 2 receptor (CB2R), including positivity, intensity, percentage, site of distribution, and immunohistochemical score, in renal cell carcinomas (RCCs) and explore their correlation with various clinicopathological aspects.

Methodology

We retrospectively obtained data and specimens from 87 patients diagnosed with RCC after partial or radical nephrectomy, and the CB1R and CB2R expression was assessed immunohistochemically on paraffin-embedded tissues. The results were statistically analyzed uni- and multi-factorial along with clinicopathological parameters.

Results

CB1R was not expressed at all, and CB2R was highly expressed in 78 (89.7%) patients with RCC. In unifactorial analysis, no statistical significance was found in any of the analyzed parameters. However, in the multifactorial analysis, we found that patients with a papillary histologic type (*P *< 0.0005) were associated with a lower likelihood of expression of the CB2R in the membranous compared with those with clear-cell and were also associated with a higher likelihood of moderate or strong expression of CB2R immunohistochemical score compared with those with clear-cell (*P *= 0.03). Patients with stage T2 (*P *= 0.010) had more enhanced expression (grade 3 CB2R intensity) compared with those with stage T1. Males (beta coefficient ± standard error [SE] 13.70 ± 7.04; *P *= 0.056) and patients with chromophobe histologic type (beta coefficient ± SE 23.45 ± 9.86; *P *= 0.020) were associated with a higher percentage of CB2R expression.

Conclusions

Our data suggest that although the CB1R was not expressed in RCCs, CB2R was expressed in almost every patient and enhanced expression was noted in correlation with specific clinicopathological aspects of the patients. Thus, following well-designed studies, especially CB2R could be used as a prognostic marker or even as a potential therapeutic target in RCC.

## Introduction

Renal cell carcinoma (RCC) represents around 3% of all cancers, the sixth most common cancer in men and tenth in women, with the highest incidence occurring in Western countries [1-3]. During the last two decades until recently, there has been an annual increase of about 2% in incidence; however, mortality rates are generally stabilizing or declining [4]. There are three main RCC histologic types: clear cell, papillary (no longer divided into types I and II), and chromophobe according to the World Health Organization (WHO) [5]. Various molecular factors and different prognostic models have been investigated in RCCs regarding prognosis. However, there is no conclusive evidence on the value of these markers for treatment selection and no conclusive evidence that one prognostic model is more accurate than another. Therefore, their routine use in clinical practice is not recommended until further validation studies have been completed [6,7]. Most cases of RCC are diagnosed incidentally and are usually treated with either partial or radical nephrectomy [8]. However, if further treatment is needed, none of the conventional adjuvant treatments, such as chemotherapy/radiotherapy, are effective in RCCs [9], and thus, targeted therapies are considered standard care [10-12].

Cannabinoids are the active components of the plant *Cannabis sativa* and are further divided into phytocannabinoids, endogenous cannabinoids, and synthetic cannabinoids [13]. They work through the activation of two G-protein-coupled receptors, the cannabinoid receptor type 1 (CB1R) and the cannabinoid receptor type 2 (CB2R), called the endocannabinoid system (ECS) within a cell [14]. If activated, a cascade of additional enzymes and nuclear factor activations appears to inhibit cancer cell proliferation and angiogenesis, demonstrating antitumor properties [15]. Consequently, an increasing number of studies aim to evaluate the expression of cannabinoid receptors in a wide variety of cancers. Promising results are already emerging in the literature, such as the downregulation of CB1R in clear-cell RCC [16,17].

We aimed to evaluate the expression of CB1 and CB2 receptors in RCCs and assess subcategories of their expression (percentage, intensity, site, etc.) in association with various clinicopathological parameters, patients' demographic data, and determine whether they can be used as predictive markers for the clinical course and prognosis, or even as therapeutic targets in the future.

## Materials and methods

We initially obtained retrospective data for a total of 96 patients diagnosed with RCC from Laiko Hospital in Athens, one of the leading Cancer Centers in Greece, and the Teaching Hospital of the Athens Medical School. Full demographic data were available for 87 of these patients, and those were included at the end of our study. These patients had undergone either radical or partial nephrectomy from January 2009 until September 2010 due to a kidney tumor and were subsequently been diagnosed with RCC. The specimens were obtained from the Pathology Laboratory of Athens Medical School, and the pathology reports of all these 87 partials or radical nephrectomies were collected. The paraffin blocks of the most appropriate slides were used, and new sections were cut and prepared for immunohistochemical analysis. They were then processed with the following kits, including stains and antibodies: (1) Vectastain Elite ABC Kit (Universal), PK-6200 (Vector Laboratories, Burlingame, CA); (2) normal horse serum (S-2000, Vector Laboratories); (3) ImmunoCruz TM goat ABC Staining System (sc-2023; Santa Cruz Biotechnology, Dallas, TX); (4) anti-CB2 (H-60, sc-25494; Santa Cruz Biotechnology); and (5) anti-CB1 (N-15, sc-10066; Santa Cruz Biotechnology).

Statistical analysis

Data were expressed as mean ± standard deviation (SD) or median and interquartile range (IQR) for quantitative variables and as frequencies (*n*) and percentages (%) for categorical variables. Unifactorial analyses were conducted using the student t-test and chi-square test or Fisher exact test to examine the relationship between the following categorical outcome variables: (1) CB2R positivity (negative vs. positive), (2) CB2R intensity (1 vs. 2 vs. 3), (3) CB2R membranous (no vs. yes), and (4) CB2R immunohistochemical score (a combination of percentage of expression with intensity) and the quantitative and qualitative demographic and clinicopathological variables, respectively. Unifactorial analyses were conducted using the Kruskal-Wallis test with the Dunn test, adjusted by the Benjamini-Hochberg FDR method for pairwise comparisons. Additionally, Spearman's correlation coefficient was employed to analyze the relationship between the quantitative outcome variable CB2R (%) and the quantitative and qualitative demographic and clinical variables, respectively. Variables in the univariate analysis were further assessed in the multifactorial binary logistic and linear regression model with the enter method to identify independent demographic and clinical predictors of the outcome variables. All assumptions of regression models (homoscedasticity, linearity, normality, and independence of error terms, as well as multicollinearity of independent variables) were examined. All tests are two-sided. The statistical significance was set at *P* < 0.05. All analyses were carried out using IBM SPSS Statistics for Windows, Version 21.00 (IBM Corp., Armonk, NY).

## Results

We included 87 patients, with a mean (SD) age of 60.7 (12.8) years, who had undergone either partial or radical nephrectomy and were diagnosed with nonmetastatic (M0) RCC. The demographics and clinical characteristics are shown in Table 1. Fifty-nine (67.8%) patients were male, and the rest were female. Regarding the histologic type of the RCCs, 55 (63.2%) patients had clear-cell carcinoma, 17 (19.5%) papillary, and 10 (11.5%) had chromophobe, and we also found five patients with unclassified histologic type. Of those, 61 (70.1%) patients had T1 disease with tumors <7 cm confined within the renal capsule, 11 (12.6%) had T2 stage with tumors < or > than 10 cm but again confined within the renal capsule, and 15 (17.2%) presented with T3 disease, involving extra-renal extension or the renal vein. The majority of patients (83, 95.4%) were diagnosed with N0 disease, with only 4 patients found to have positive lymph nodes (N1). Regarding the grade, we found 14 (16.1%) patients with grade 1 and 40 (46%), 27 (31%), and 6 (6.9%) patients with grades 2, 3, and 4, respectively.

**Table 1:** Demographic, clinical characteristics, and CB2R expression characteristics. CB2R, cannabinoid type 2 receptor

Characteristics		n	%
Histologic type	Clear cell	55	63.2
	Papillary	17	19.5
	Chromophobe	10	11.5
	Unclassified	5	5.7
Grade	1	14	16.1
	2	40	46.0
	3	27	31.0
	4	6	6.9
Gender	Female	28	32.2
	Male	59	67.8
Stage Τ	1	61	70.1
	2	11	12.6
	3	15	17.2
Stage Ν	0	83	95.4
	1	2	2.3
	2	2	2.3
CB2R positivity	Negative	9	10.3
	Positive	78	89.7
CB2R intensity	1	32	41.0
	2	34	43.6
	3	12	15.4
Age	Mean ± SD (min-max): 60.7 ± 12.8 (25-85)		
CB2R (%)	Median (IQR) (min-max): 35.0 (51.0) (7-95)		

Paradoxically, CB1R was not expressed in any of the specimens, suggesting either a malfunction of the antibody or that this receptor was not expressed in RCCs. Consequently, our results and analysis were focused solely on CB2R.

Table 1 also provides information regarding the expression of CB2R and its sub-characteristics. In 78 (89.7%) patients, CB2R was expressed, which means in most patients with RCC, this receptor was expressed. In 32 (41%) patients, the intensity of the expression was 1, in 34 (43.6%) the intensity was 2, and in 12 (15.4%) the intensity was 3. Finally, the median (IQR) (min-max) of the CB2R was 35%.

Table 2 presents the unifactorial analysis of the demographic and clinical characteristics of the patients with RCC about CB2R positivity and intensity. Table 3 provides information about the unifactorial analysis of demographic and clinical characteristics of the percentage of CB2R expression and the immunohistochemical score. The immunohistochemical score used for our study is the combination of percentage and intensity scores into a final expression score, known as the immunoreactive score (IRS) scoring method. IRS is calculated as follows: Final expression score = percentage of positive cells multiplied by the staining intensity. This results in a final score ranging from 0 to 12. The final expression score was divided into four categories (0-1 = no expression, 2-3 = weak expression, 4-8 = moderate expression, and 9-12 = strong expression).

**Table 2:** Unifactorial analysis of demographic and clinical characteristics in relation to CB2R positivity and intensity. All categorical variables were presented as frequencies (%). CB2R, cannabinoid type 2 receptor

Variables		CB2R positivity		*P*-value	CB2R intensity			*P*-value
		Negative (*n *= 9)	Positive (*n *= 78)		1 (*n *= 32)	2 (*n *= 34)	3 (*n *= 12)	
Age (years), mean ± SD		54.11 ± 10.34	61.41 ± 12.86	0.105	60.81 ± 13.91	62.18 ± 12.00	60.83 ± 13.32	0.901
Histologic type	Clear cell	7 (12.7)	48 (87.3)	0.493	23 (47.9)	18 (37.5)	7 (14.6)	0.524
	Papillary	2 (11.8)	15 (88.2)		5 (33.3)	7 (46.7)	3 (20.0)	
	Chromophobe	0 (0.0)	10 (100.0)		2 (20.0)	6 (60.0)	2 (20.0)	
Grade	1	2 (14.3)	12 (85.7)	0.580	3 (25.0)	6 (50.0)	3 (25.0)	0.462
	2	5 (12.5)	35 (87.5)		18 (51.4)	13 (37.1)	4 (11.4)	
	3	2 (6.1)	31 (93.9)		11 (35.5)	15 (48.4)	5 (16.1)	
Gender	Male	7 (11.9)	52 (88.1)	0.712	17 (32.7)	25 (48.1)	10 (19.2)	0.088
	Female	2 (7.1)	26 (92.9)		15 (57.7)	9 (34.6)	2 (7.7)	
Stage Τ	1	5 (8.2)	56 (91.8)	0.555	23 (41.1)	27 (48.2)	6 (10.7)	0.083
	2	2 (18.2)	9 (81.8)		4 (44.4)	1 (11.1)	4 (44.4)	
	3	2 (13.3)	13 (86.7)		5 (38.5)	6 (46.2)	2 (15.4)	

**Table 3:** Unifactorial analysis of demographic and clinical characteristics in relation to CB2R (%) and immunohistochemical score. Categorical variables for CB2R immunohistochemical score and CB2R (%) were presented as frequencies (%) and median (IQR ), respectively. CB2R, cannabinoid type 2 receptor; IQR, interquartile range

Variables		CB2R (%)	*P*-value	CB2R immunohistochemical score		*P*-value
				No-weak (*n* = 45)	Moderate-strong (*n *= 37)	
Age; Spearman correlation coefficient		-0.024	0.832	59.53 ± 13.07	61.98 ± 12.50	0.378
Histologic type	Clear cell	32.5 (49.0)	0.095	33 (60.0)	22 (40.0)	0.211
	Papillary	30.0 (60.0)		9 (52.9)	8 (47.1)	
	Chromophobe	67.5 (51.0)		3 (30.0)	7 (70.0)	
Grade	1	32.5 (55.0)	0.547	7 (50.0)	7 (50.0)	0.329
	2	30.0 (50.0)		25 (62.5)	18 (37.5)	
	3	54.0 (50.0)		15 (45.5)	18 (54.5)	
Gender	Male	45.0 (55.0)	0.100	19 (67.9)	9 (32.1)	0.107
	Female	20.0 (42.0)		28 (47.5)	31 (52.2)	
Stage Τ	1	32.5 (44.0)	0.338	33 (54.1)	28 (45.9)	0.998
	2	80.0 (66.0)		6 (54.5)	5 (45.5)	
	3	60.0 (55.0)		8 (53.3)	7 (46.7)	

Our purpose was to examine whether the patients' demographics, but most importantly, the clinicopathological characteristics of the RCCs, would correlate with the expression of the CB2 receptor and further subcategories of expression, such as intensity, percentage of expression, and their combination. This examination aimed to support the suggestion that CB2R is clinically important for RCCs. However, there were no statistically significant associations between age, histologic type, grade, stage, and gender and the assessed quantitative and qualitative outcomes: CB2R positivity, intensity, percentage of expression, and immunohistochemical score (Tables 2-3).

Tables 4-6 present a multifactorial analysis. Multiple logistic regression models with the enter method (all variables in the unifactorial analysis included in the model) were employed to examine the effect of demographic and clinical variables on qualitative outcomes (CB2R positivity, percentage of expression, distribution in cytoplasm and membrane, and immunohistochemical score). All models satisfied all assumptions of logistic regression analysis, and the results were as follows: The model was not statistically significant Χ2 (8) = 8.98, *P *= 0.344, accounting for 20% (Nagelkerke R2) of the variance of BC2R positivity. Νone of the factors had a statistically significant effect on the BC2R positivity (Table 4). The model was not statistically significant Χ2 (8) = 12.17, *P *= 0.144. The model explained 25.3% (Nagelkerke R2) of the variance of BC2R cytoplasmic distribution. Νone of the factors had a statistically significant effect on the CB2R cytoplasmic distribution (Table 5). The model was statistically significant Χ2 (8) = 24.65, *P* = 0.02. The model explained 39% (Nagelkerke R2) of the variance of BC2R membranous distribution. Patients with the papillary histologic type (odds ratio [OR] 0.03, 95% confidence interval [CI] 0-0.16; *P *< 0.0005) were associated with a lower likelihood of expression of CB2R in the membranous compared with those with clear cells (Table 5). The model was not statistically significant Χ2 (8) = 10.397, *P *= 0.239, accounting for 22.4% (Nagelkerke R2) of the variance of BC2R intensity. Only patients with stage T2 (OR 13.55, 95% CI 1.87-97.96; *P* = 0.010) were associated with a higher likelihood of grade 3 CB2R intensity (higher intensity grade) compared with those with stage T1, but not with those with stage T3 (Table 4). The model was not statistically significant Χ2 (8) = 8.89, *P* =0.351, accounting for 14% (Nagelkerke R2) of the variance of the BC2R immunohistochemical score. Only patients with the papillary histologic type (OR 5.9, 95% CI 1.2-30.2; *P* = 0.033) were associated with a higher likelihood of moderate or strong expression of CB2R immunohistochemical score compared with those with clear-cell type (Table 5).

**Table 4:** Multifactorial analysis of demographic and clinical characteristics in relation to CB2R positivity and intensity. OR, odds ratio; CI, confidence interval; CB2R, cannabinoid type 2 receptor

	Reference category	CB2R positivity				CB2R intensity			
		OR	95% CI		*P*-value	OR	95% CI		*P*-value
Age	-	1.06	1.00	1.13	0.079	1.02	0.96	1.08	0.587
Gender (male )	Female	0.64	0.11	3.73	0.617	3.45	0.51	23.36	0.205
Grade					0.362				0.378
2	1	1.04	0.16	6.92	0.970	0.25	0.03	1.76	0.163
3		0.63	0.35	50.21	0.261	0.39	0.05	2.93	0.357
Stage Τ					0.145				0.035
2	1	0.19	0.03	1.22	0.080	13.55	1.87	97.96	0.010
3		0.18	0.02	1.69	0.134	2.25	0.27	18.86	0.454
Histologic type					0.890				0.873
Papillary	Clear-cell	1.08	0.17	6.75	0.932	1.41	0.26	7.62	0.687
Chromophobe		1.84	0.15	22.25	0.630	1.52	0.19	12.42	0.695

**Table 5:** Multifactorial analysis of demographic and clinical characteristics in relation to CB2R cytoplasmic distribution, membranous distribution, and immunohistochemical score. OR, odds ratio; CI, confidence interval; CB2R, cannabinoid type 2 receptor

	Reference category	CB2R cytoplasmic distribution				CB2R membranous				CB2R immunohistochemical score			
		OR	95% CI		*P*-value	OR	95% CI		*P*-value	OR	95% CI		*P*-value
Age	-	0.97	0.92	103	0.367	1.01	0.96	1.05	0.821	1.03	0.99	1.07	0.190
Gender (male)	Female	3.59	0.86	15.00	0.080	1.89	0.54	6.55	0.317	2.56	0.83	7.89	0.103
Grade					0.093				0.621				0.639
2	1	0.66	0.10	4.40	0.669	0.65	0.08	4.99	0.678	0.61	0.16	2.26	0.456
3		5.01	0.46	54.94	0.187	0.40	0.05	3.33	0.397	0.94	0.22	3.98	0.928
Stage Τα					0.946				0.539				0.947
2	1	1.38	0.11	16.84	0.802	0.55	0.10	3.00	0.487	1.02	0.24	4.33	0.974
3		0.83	0.11	6.18	0.855	0.45	0.10	2.14	0.313	0.80	0.19	3.29	0.752
Histologic type					0.374				<0.0005				0.053
Papillary	Clear cell	3.83	0.39	37.48	0.249	0.03	0.0	0.16	<0.0005	1.12	0.35	3.57	0.854
Chromophobe		3.03	0.27	33.74	0.368	0.65	0.14	3.08	0.588	5.90	1.15	30.23	0.033

**Table 6:** Multifactorial analysis of demographic and clinical characteristics in relation to CB2R (%). SE, standard error; CB2R, cannabinoid type 2 receptor

	Reference category	Beta	SE	*P*-value
Constant	-	-34.47	27.15	0.209
Age	-	0.24	0.26	0.372
Gender (male)	Female	13.70	7.04	0.056
Grade (3)	1-2	1.00	6.93	0.887
Stage Τ ( 2-3 )	1	9.48	7.61	0.217
Histologic type (chromophobe)	Clear cell or papillary	23.45	9.86	0.020

A multiple linear regression model with the enter method was employed to examine the contribution of demographic and clinical variables to CB2R (%). Regression analysis accounted for 14% of the variance in CB2R (%). Males (beta coefficient ± SE 13.70 ± 7.04; *P* = 0.056), and patients with chromophobe histologic type (beta coefficient ± SE 23.45 ± 9.86; *P* = 0.020) were associated with a higher percentage of CB2R (Table 6).

## Discussion

In this study, we described that while CB1R was not expressed at all, CB2R was expressed in the vast majority of patients with RCC. Thus, it could have served as a potential future prognostic factor or therapeutic target. Specifically, we observed an association between males and patients with chromophobe RCC, showing a higher percentage of CB2R expression. Additionally, an association was found between T2 disease and a more intense expression of the receptor. Finally, it appeared that the combination of expression percentage and stain intensity (referred to as IRS) was higher in papillary RCCs. These findings were reported for the first time in a study and could be involved in a prognostic model in the future. Nevertheless, a nonsignificant correlation between CB2R expression and clinicopathological parameters was noted. Our study had several limitations. First, the sample size was relatively small (*n* = 87), and none of the patients expressed the CB1R. Second, we did not include a control group consisting of healthy patients to assess whether they also expressed the receptors. Additionally, the study design was retrospective and single-centered, leading to heterogeneity in patients' characteristics. Moreover, for the multifactorial analysis, we dealt with numerous categories, each having quite a few grades, and a relatively small number of cases. This situation may lead to potentially unreliable conclusions.

Cannabinoids have been found to have antitumor effects, both in vitro and in vivo, by mediating different signaling pathways and biological processes that play a significant role in tumorigenesis [18], including the induction of apoptosis, the inhibition of tumor cell proliferation, anti-angiogenic effects, and activation of the immune system [14,19]. These properties are likely attributed to their role in endocannabinoid signaling pathways involved in cancer processes such as the MEK-extracellular signal-regulated kinase signaling cascade, and the adenylyl cyclase, cyclic AMP-protein kinase-A pathway [20]. The upregulation and downregulation of CB receptors appear to play a significant role in cancerous processes. Additionally, the expression of these receptors in malignant tissues holds potential as prognostic markers. However, there is still a lack of studies with a high level of evidence concerning the correlation between CB receptor expression and specific clinicopathological characteristics in these patients [21].

Specific cancers, such as oral squamous cell carcinoma, pancreatic carcinoma, hepatocellular carcinoma, and prostatic carcinoma, have shown upregulation of CB receptors. Interestingly, these receptors were not expressed in nearby normal tissues [22,23]. On the other hand, Caffarel et al. demonstrated that CB1R is downregulated in breast cancer cells of women [24]. CB1R upregulation has also been reported in cancers such as prostatic adenocarcinoma, hepatocellular carcinoma, and pancreatic ductal adenocarcinoma [17,25], while CB1R downregulation was demonstrated in colorectal carcinoma compared to healthy organ tissue [26]. Thyroid gland cancer exhibited enhanced expression of the CB receptors, particularly CB2R, correlated with patients' clinical characteristics crucial for the disease course and prognosis [18]. Another recent study provides evidence that CB1R and CB2R may play a role in the pathophysiological aspects of the mobile tongue SCC [27]. In terms of prognosis, head and neck squamous cell carcinomas with significant CB2R expression were associated with shorter survival [28]. In 2005, Sarfaraz et al. showed that patients with more advanced prostate cancer (higher Gleason score, larger tumor size, or metastatic at diagnosis) had CB1R overexpressed and thus associated with worst prognosis [25].

To date, only a few studies have examined the correlation between CB receptors in the kidney and RCC. Many years ago, the effects of cannabinoids on renal cancer cells were tested in vitro using cell cultures [29]. A few years later, in 2008, Khan et al. showed that CB2R can indeed inhibit RCC cells in vitro [9]. Another study revealed that CB1R appeared to be downregulated in clear-cell RCC [17]. CB1R showed intense positive immunostaining in chromophobe RCC and renal oncocytoma and thus could be used as a diagnostic tool in the differential diagnosis of RCCs [30].

## Conclusions

CB1R was not expressed in our study, either due to antibody malfunction or because it was not expressed in the RCCs. In contrast, CB2R was highly positive in 89.7% of the cancers. Initially, the unifactorial analysis did not reveal any significant association between patients' demographic data, clinicopathological characteristics, and the expression of CB2R. However, in the multifactorial analysis, males and patients with the chromophobe histologic type, which typically has favorable prognoses, were associated with higher percentages (13.7% and 23.5% higher, respectively) of CB2R expression. Moreover, patients with T2 disease were found to have a 13.6 times higher intensity grade of CB2R expression compared to the T1 patients, and papillary RCCs correlated with six times higher expression of the CB2R immunohistochemical score compared to clear-cell malignancies. Our findings imply that CB2R could potentially be used as a predictive tissue marker for RCCs and even represent a potential therapeutic strategy. For conclusive results, studies with higher levels of evidence are needed in the future.
